# Ninjurin1 Deletion in NG2-Positive Pericytes Prevents Microvessel Maturation and Delays Wound Healing

**DOI:** 10.1016/j.xjidi.2022.100141

**Published:** 2022-07-07

**Authors:** Risa Matsuo, Mari Kishibe, Kiwamu Horiuchi, Kohei Kano, Takamitsu Tatsukawa, Taiki Hayasaka, Maki Kabara, Shin Iinuma, Ryoji Eguchi, Satomi Igawa, Naoyuki Hasebe, Akemi Ishida-Yamamoto, Jun-ichi Kawabe

**Affiliations:** 1 Department of Biochemistry, Asahikawa Medical University, Asahikawa, Japan; 2 Department of Dermatology, Asahikawa Medical University, Asahikawa, Japan; 3 Division of Cardiovascular, Respiratory and Neurology, Department of Medicine, Asahikawa Medical University, Asahikawa, Japan; 4 Department of Vascular Surgery, Asahikawa Medical University, Asahikawa, Japan; 5 Department of Cardiovascular Regeneration and Innovation, Asahikawa Medical University, Asahikawa, Japan

**Keywords:** 3D, three-dimensional, EC, endothelial cell, KC, keratinocyte, KO, knockout, Ninj1, Ninjurin-1, PC, pericyte, Tam, tamoxifen, VSMC, vascular smooth muscle cell, WT, wild-type

## Abstract

The formation of mature vasculature through angiogenesis is essential for adequate wound healing, such that blood-borne cells, nutrients, and oxygen can be delivered to the remodeling skin area. Neovessel maturation is highly dependent on the coordinated functions of vascular endothelial cells and perivascular cells, namely pericytes (PCs). However, the underlying mechanism for vascular maturation has not been completely elucidated, and its role in wound healing remains unclear. In this study, we investigated the role of Ninjurin-1 (Ninj1), a new molecule mediating vascular maturation, in wound healing using an inducible PC-specific *Ninj1* deletion mouse model. Ninj1 expression increased temporarily in NG2-positive PCs in response to skin injury. When tamoxifen treatment induced a decreased Ninj1 expression in PCs, the neovessels in the regenerating wound margins were structurally and functionally immature, but the total number of microvessels was unaltered. This phenotypic change is associated with a reduction in PC-associated microvessels. Wound healing was significantly delayed in the NG2-specific *Ninj1* deletion mouse model. Finally, we showed that Ninj1 is a crucial molecule that mediates vascular maturation in injured skin tissue through the interaction of vascular endothelial cells and PCs, thereby inducing adequate and prompt wound healing.

## Introduction

Wound healing is a complex process in which the skin regenerates itself after an injury. It involves three overlapping phases: blood clotting and inflammation, proliferation, and remodeling (Schäfer and Werner, 2008), for which a dynamic angiogenic process is crucial. During skin damage, first, hemostasis is initiated by intravascular platelets, which ultimately forms a clot; stops the bleeding; and causes the accumulation of inflammatory cells, including macrophages. Second, the proliferative phase is commenced, and it includes granulation tissue formation and re-epithelialization of the skin. Incidentally, there is an intensive formation of microvessels within the wound area and a simultaneous accumulation of proliferated fibroblasts to form the granulation tissue and produce a new extracellular matrix. Moreover, activated keratinocytes (KCs) around the wound margin migrate over the provisional matrix deposited to cover the wound.

In the early wound healing phase, although most of the neovasculatures, that is, newly formed capillaries, are immature and not effectively perfused, they consequently mature to form functional and structurally stabilized microvessels that bring nutrients and oxygen to the wound area and help in the regeneration of peripheral nerves, all of which are essential for appropriate wound healing (Cañedo-Dorantes and Cañedo-Ayala, 2019). Finally, there is gradual regression of blood vessels in the remodeling phase until their density resembles normal, uninjured skin. This phase is characterized by selective apoptosis of excess, newly formed microvessels along with the gradual regression and fibrosis of the granulation tissue; in fact, this is important to ensure the completion of a normal wound healing process and avoid excessive scar formation (DiPietro, 2016).

Dynamic changes in the vasculature, including angiogenesis, vascular maturation, and regression, closely depend on the presence of perivascular cells, including pericytes (PCs) (Thomas et al., 2017). In the initial angiogenic phase, PCs and endothelial cells (ECs) perform a crucial role: the PCs get detached from pre-existing vessels and provide an environment for the growth of the EC sprouts through degradation of extracellular matrix and release of angiogenic GFs (Armulik et al., 2011; Kelly-Goss et al., 2014). The angiogenic maturation phase is characterized by the recruitment of PCs into EC tubes, thereby providing them with structural stability and functional abilities (Gaengel et al., 2009). When PCs are lost from existing vessels owing to pathophysiological conditions, such as diabetes mellitus, the vessels become unstable and/or undergo regression (Hall, 2006; Warmke et al., 2016). Although the angiogenic effects of PCs have been documented (Armulik et al., 2011; Huang, 2020), the underlying mechanisms that regulate their actions in vascular maturation and stabilization have not been completely clarified.

The nerve injury‒induced protein, Ninjurin-1 (Ninj1), a cell surface adhesion molecule with homophilic binding activity, was originally identified as a protein that gets expressed in peripheral nerve tissues in response to nerve injury (Araki et al., 1997; Araki and Milbrandt, 1996). However, it was established later that Ninj1 is expressed by various tissues or cells, and it is involved in diverse pathophysiological conditions, including inflammation and neural regeneration (Choi et al., 2018; Lee et al., 2010; Tomita et al., 2019; Yin et al., 2014). Recently, we showed that Ninj1 in vascular cells plays a crucial role in angiogenesis, thereby mediating the formation of mature blood vessels through the association of ECs and PCs (Matsuki et al., 2015). Moreover, Ninj1 is temporally expressed in response to ischemia. In fact, an NG2-specific *Ninj1-*deletion mouse model showed that Ninj1 in the PCs contributes to the recovery of blood flow in case of hind limb ischemia through the formation of functional vessels (Minoshima et al., 2018). In addition, Ninj1 plays an essential role in the formation of mature microvessels within the thickened walls of injured vasculature, thereby contributing to vascular remodeling (Horiuchi et al., 2021). Even though these observations suggest that Ninj1 is a crucial molecule for the maturation of neovessels under various pathophysiological conditions, the role of Ninj1 in angiogenesis concerning skin wound healing remains unclear. Hence, in this study, we performed an imaging analysis of the three-dimensional (3D) architecture of the skin microvessels and investigated the roles of Ninj1 on angiogenesis and wound healing in an NG2^+^ cell‒specific *Ninj1*-deletion mouse model.

## Results

### NG2 is a marker for PCs/vascular smooth muscle cells of cutaneous microvessels

The PCs are defined by their abluminal spatial relationship with vascular ECs and their specific markers (Díaz-Flores et al., 2009). PDGFRβ and NG2 have emerged as cellular markers that confirm the presence of PCs/vascular smooth muscle cells (VSMCs) within the microvasculature (Minoshima et al., 2018; Vanlandewijck et al., 2018). However, these markers are not exclusively expressed by these cells. To examine whether NG2 is a reliable marker for PCs/VSMCs in the skin, we analyzed the occurrence of NG2^+^ cells within the skin of transgenic mice expressing *DsRed* under the NG2 promoter (NG2-DsRed mice).

**Figure 1:**
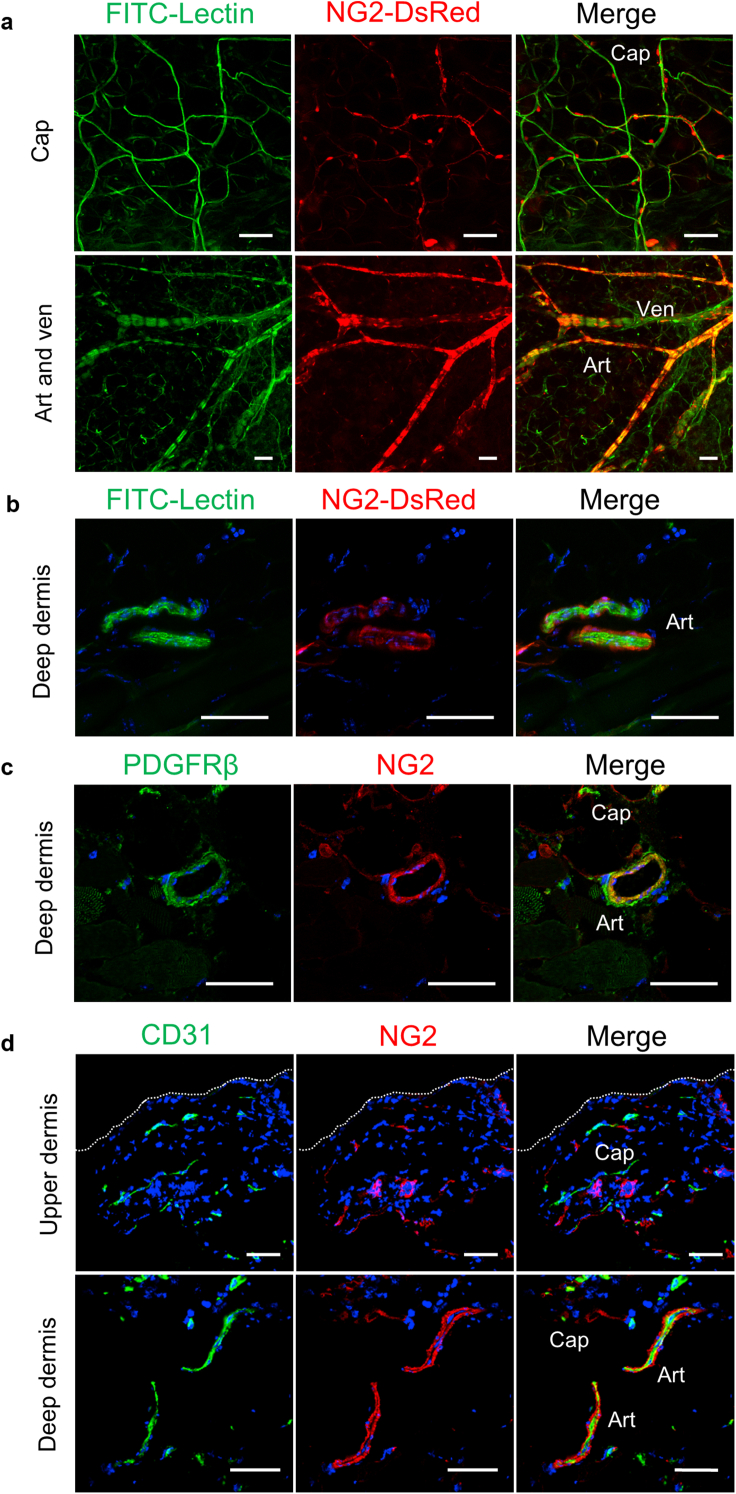
**NG2 is a marker for pericytes/vascular smooth muscle cells of cutaneous microvessels.** Mice expressing DsRed under the NG2 promoter (NG2-DsRed mice) were injected with FITC-lectin through tail veins to visualize blood vessels. (**a**) Whole-mount images of the normal ear skin of NG2-DsRed mice. Caps and Art/Ven perfused with FITC-lectin. (**b**) Perfusion labeling with FITC-lectin in the dermis of NG2-DsRed mouse skin sections. (**c**) Immunostaining for NG2 and PDGFRβ in wild-type mice. (**d**) Wild-type mouse skin sections were immunostained for CD31 and NG2. The dotted lines indicate the border between the epidermis and dermis. Bars = 50 μm. Art, arteriole; Cap, capillary; Ven, venule.

In the whole-mount skin preparations of the pinna of the mice, NG2^+^ cells were adjacent to FITC-lectin‒labeled capillary ECs. The NG2^+^ cells were also colocalized with larger blood vessels, namely arterioles and venules (Figure 1a). On the basis of the short-axis view of arterioles in the deep dermal layers of normal skin, NG2^+^ cells were present around the ECs (Figure 1b). To further characterize NG2-expressing cells, wild-type (WT) mouse skin sections were stained with anti-NG2 and anti-PDGFRβ, another PCs/VSMCs marker. PDGFRβ was expressed in NG2^+^ PCs/VSMCs of capillaries and arterioles (Figure 1c). Immunostaining of skin sections of WT mice using anti-NG2 revealed the presence of NG2^+^ cells around CD31^+^ ECs, similar to that observed in NG2-DsRed mice (Figure 1d). Therefore, NG2 is a reliable marker for PCs/VSMCs in cutaneous microvessels.

### PCs are abundantly distributed to cover new blood vessels around the wound margins

**Figure 2:**
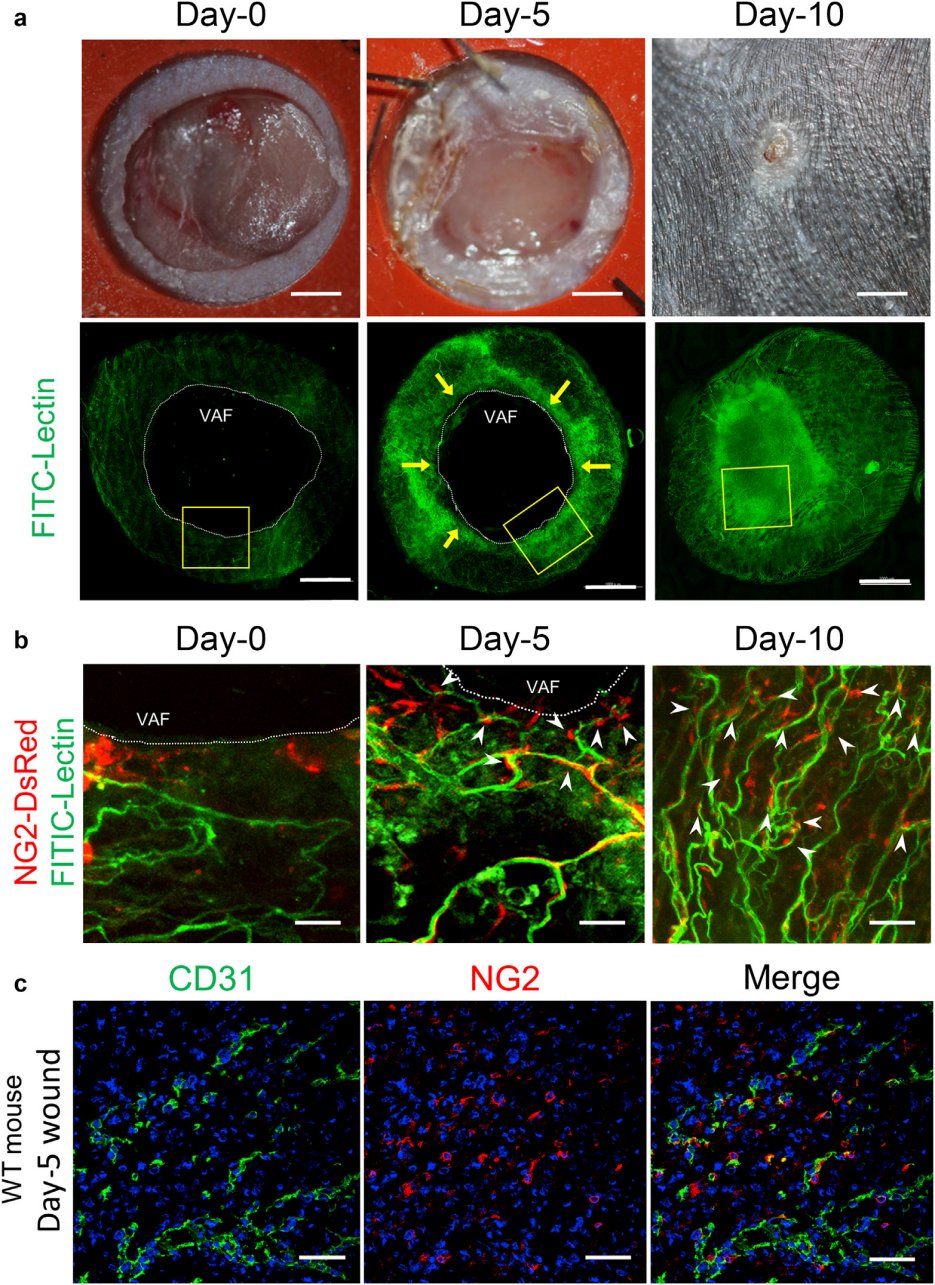
**Neovasculatures and location of pericytes along the skin wound margins.** Full-thickness skin wounds were created on the backs of NG2-DsRed mice. (**a**) Representative photographs of skin wounds on days 0, 5, and 10 and whole-mount, fluorescent images labeled with FITC-lectin corresponding to each picture. Arrows indicate the VAF, in which neovascularization occurs along the wound margins. (**b**) Higher magnification of the yellow-boxed area in **a**. White arrows indicate NG2^+^ pericytes adjacent to lectin-stained neovessels. (**c**) Skin wound sections of wild-type mice (day 5 after wounding) were immunostained for CD31 and NG2. Bars = 1 mm for **a** and 50 μm for **b** and **c**. VAF, vascular advancing front; WT, wild-type.

**Figure 3:**
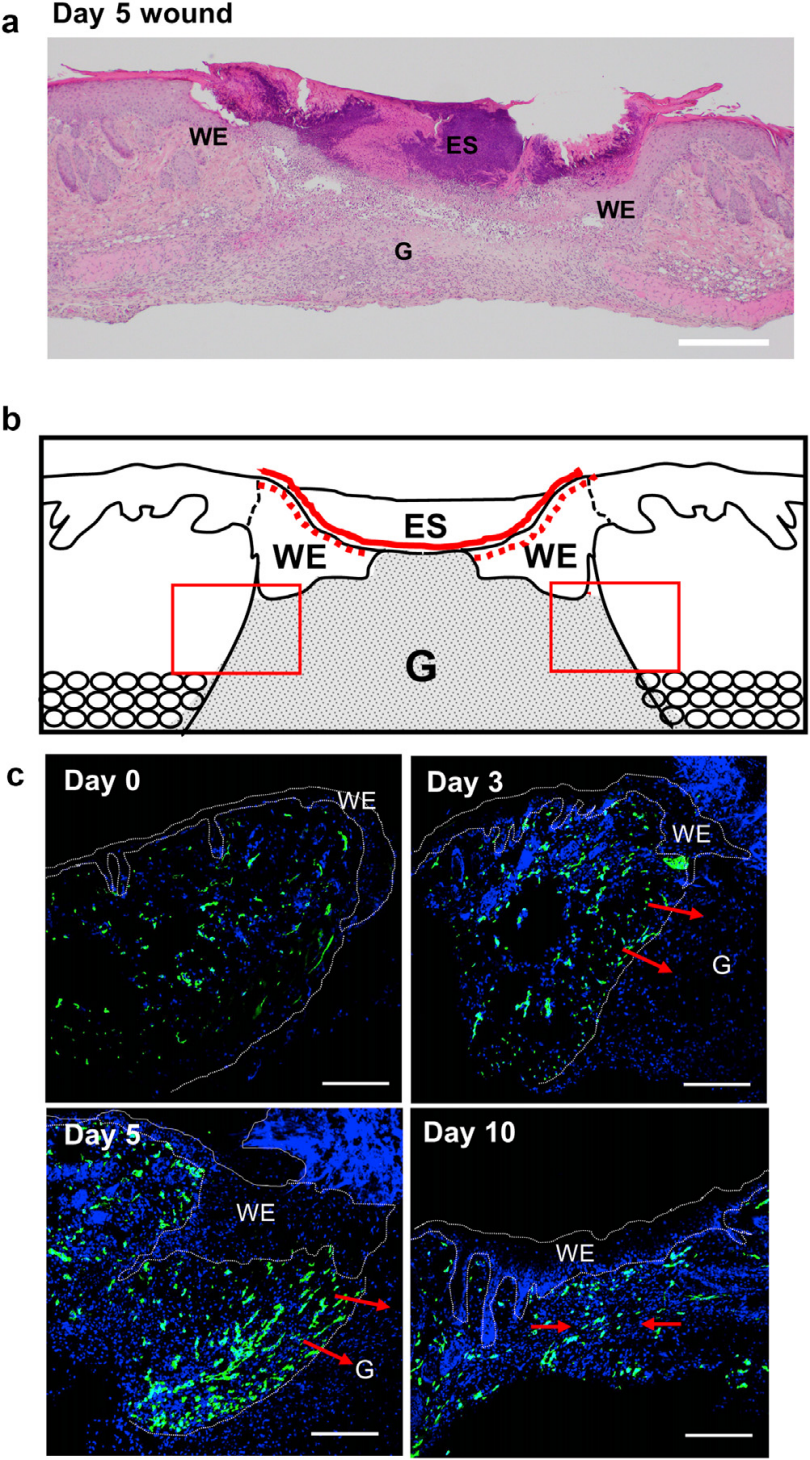
**Analysis of angiogenesis in the skin wound.** (**a**) Representative H&E-stain view on day 5 after injury. (**b**) Schematic drawing of day-5 wound skin. The wound length is highlighted with a red line. WE is highlighted with red dotted lines. Neovascularization occurs in the indicated square frame regions within the wound margin area. (**c**) Immunofluorescent staining with CD31 antibody representing angiogenesis during the wound healing process in wild-type mice (days 0, 3, 5, and 10). Angiogenesis is gradually enhanced in parallel with skin regeneration of the wound margin and is most prominent on day 5 after injury, with new blood vessels invading the granulation tissue from the wound margin. On day 10 after injury, blood vessels on both sides are merged. Bar = 200 μm. ES, eschar; G, granulation tissue; WE, wound epithelium.

To examine the behavior of PCs during skin wound healing, we observed 3D images of angiogenesis and the location of NG2^+^ cells within wounds using whole-mount transparent samples from NG2-DsRed mice. On day 0 (i.e., on the day of the surgery), FITC-lectin‒perfused blood vessels were disrupted along the wound margins. On day 5 after surgery, neovessels grown from the wound margins were observed (Figures 2a). Wound-resident NG2^+^cells increased in parallel with the formation of neovessels, and they were located around the microvessels (Figure 2b). On day 10, the wound area was mostly closed macroscopically (Figure 2a), and it was completely covered by epithelium, along with abundant, mature blood vessels, to which NG2^+^PCs were attached (Figure 2b). To confirm these results, we performed immunostaining of NG2^+^ cells in the wound sections of WT mice. On day 5, numerous NG2^+^ cells were observed along the wound margin, and they were located around and adhered to CD31^+^ ECs (Figure 2c). Angiogenesis was gradually enhanced in parallel with skin regeneration of the wound margin and was most prominent on day 5 after injury, with new blood vessels invading the granulation tissue from the wound margin (Figure 3).

### Ninj1 is expressed in PCs/VSMCs of cutaneous blood vessels

**Figure 4:**
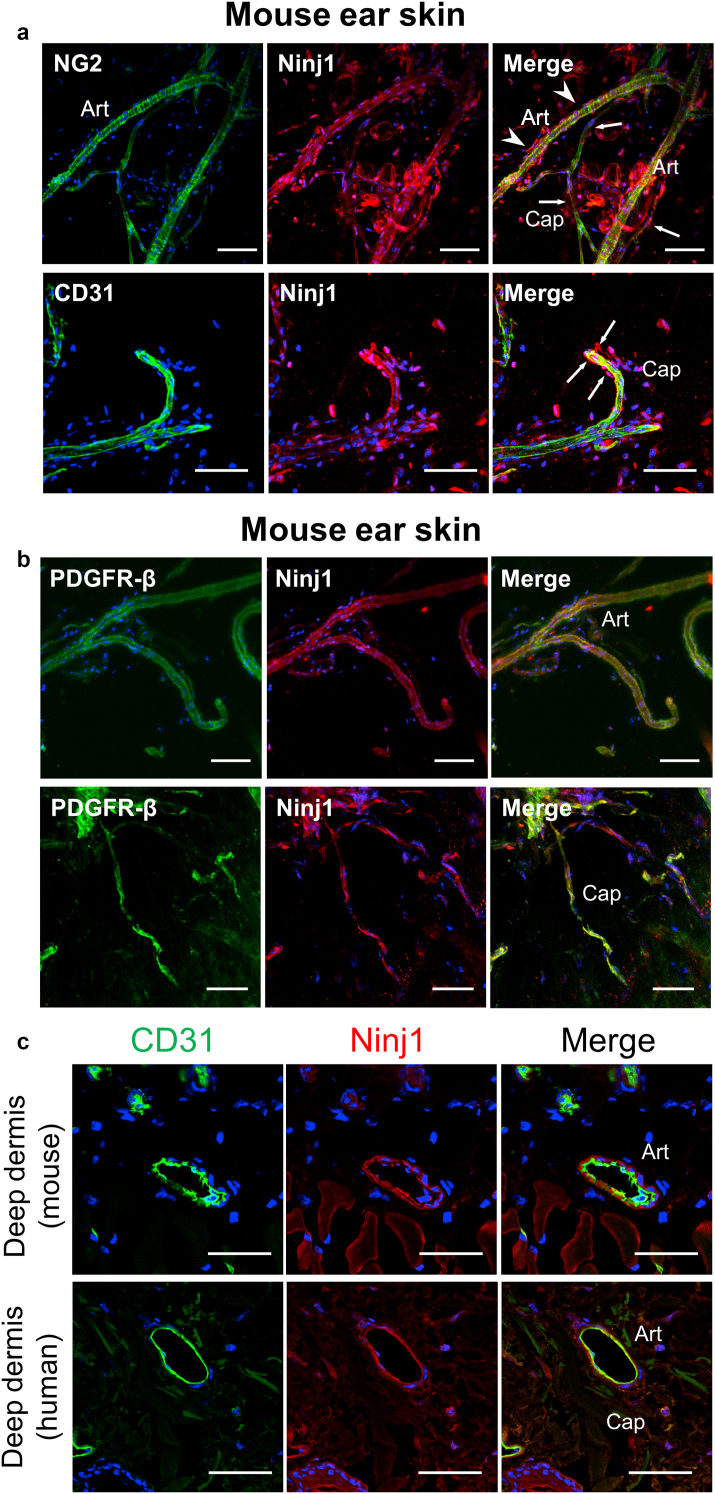
**Expression of Ninj1 in pericytes/vascular smooth muscle cells of cutaneous microvessels.** (**a, b**) Images of whole-mount, immunostained sections of normal ear skin samples of wild-type mice showing Ninj1 expression in vascular endothelial cells (CD31^+^) and pericytes. (**c**) Double immunostaining for CD31 and Ninj1 in the dermis of normal skin samples of mice or humans. Ninj1 is expressed in pericytes and vascular smooth muscle cells of Caps and Arts. Bars = 50 μm. Art, arteriole; Cap, capillary; Ninj1, Ninjurin-1.

The 3D imaging of whole-mount, transparent, immunostained skin samples showed that Ninj1 was expressed in vascular cells, both CD31^+^ ECs and NG2- or PDGFRβ-positive PCs (Figure 4a and b). We observed thin and immunostained skin sections of mice and humans and confirmed that Ninj1 was expressed in the cells adjacent to the EC layers in arterioles and capillaries (Figure 4c). The expression of Ninj1 in PCs/VSMCs of cutaneous blood vessels is similar to that in the vasculature of different tissues, including skeletal muscles and larger vessels (Horiuchi et al., 2021; Minoshima et al., 2018).

### The expression of Ninj1 is enhanced around blood vessels during wound healing

**Figure 5:**
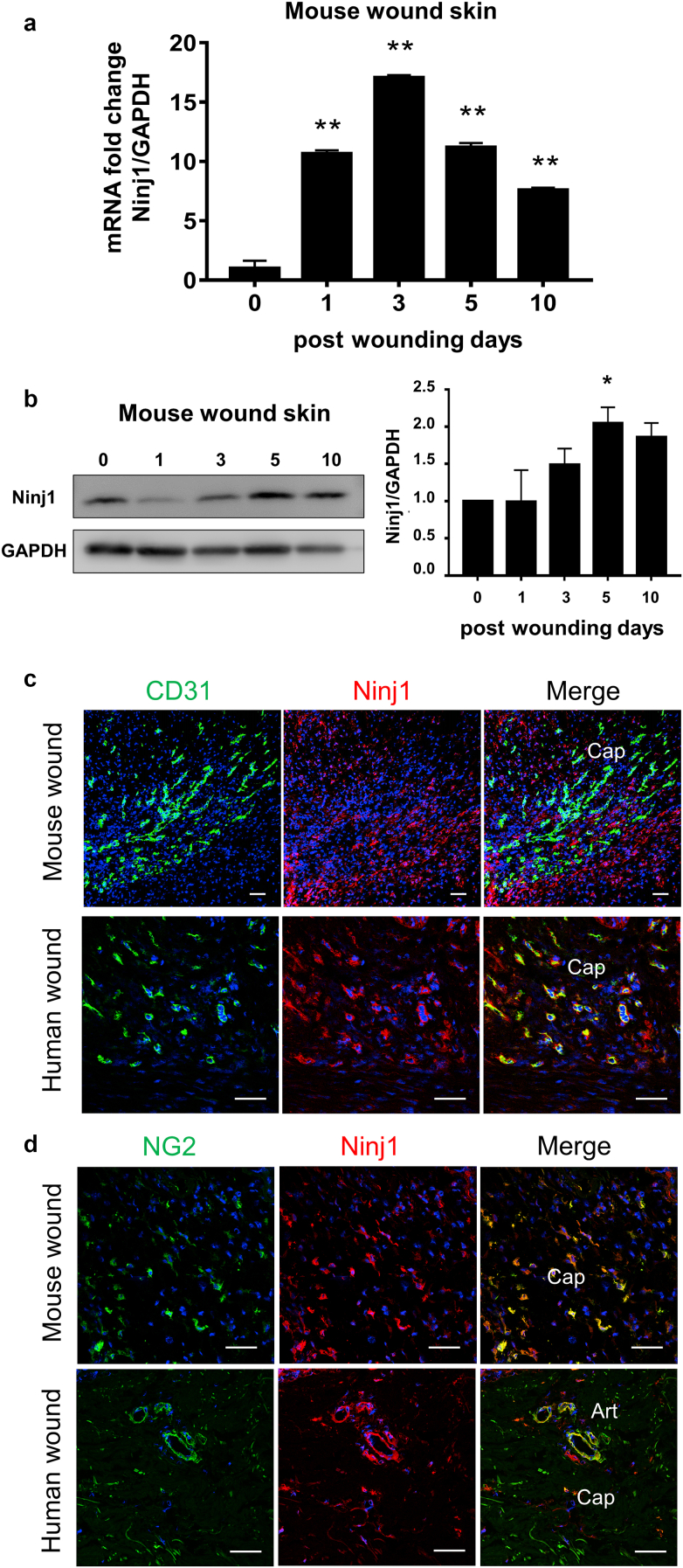
**Expression of Ninj1 in vasculature is temporarily increased during wound healing.** (**a**) *Ninj1* expression in mouse wound skin estimated by RT-qPCR at days 0, 1, 3, 5, and 10. Data are expressed as mean ± SEM. ∗∗*P* < 0.01 versus control; n = 3. (**b**) Ninj1 expression in mouse wound skin assessed by western blotting at indicated time points. The densitometric quantification is shown. Data are expressed as mean ± SEM. ∗*P* < 0.05 versus day 0; n = 3. Immunostaining for (**c**) CD31 and Ninj1 and (**d**) NG2 and Ninj1 using wound skin section of wild-type mice and humans. The representative image of human skin is from a diabetic leg ulcer (persistent for >28 days). Ninj1 expression enhanced in pericytes and VSMCs of Caps and Arts. Bars = 50 μm. Art, arteriole; Cap, capillary; Ninj1, Ninjurin-1; VSMC, vascular smooth muscle cell.

To examine whether Ninj1 expression is induced during wound healing, we subjected the skin tissue samples to a RT-qPCR and western blotting analyses. *Ninj1* mRNA expression significantly increased in response to skin wounds. It peaked on day 3 after wounding (Figure 5a), whereas Ninj1 protein expression increased and reached its peak on day 5 after injury (Figure 5b), thereby indicating that Ninj1 expression was induced during skin wound healing.

**Figure 6:**
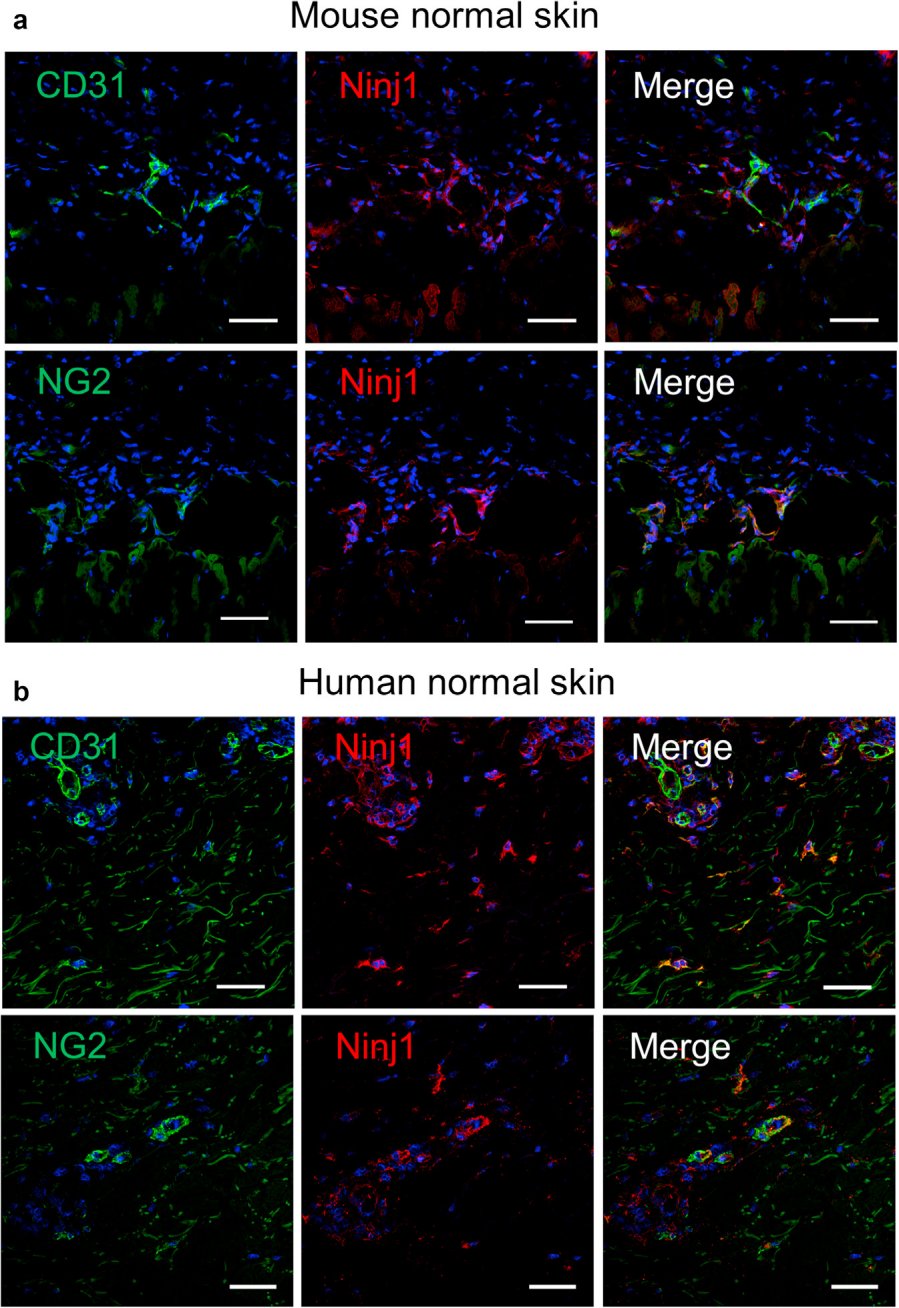
**Expression of Ninj1 in nonwound skin sections.** Double staining for CD31 and Ninj1 or NG2 and Ninj1 in normal (**a**) wild-type mice and (**b**) human skin. Bars = 50 μm. Ninj1, Ninjurin-1.

To confirm the localization of expressed Ninj1, we immunostained mouse and human skin wound sections for Ninj1 and CD31. In the mouse and human nonwound skin samples, Ninj1 expression was observed around CD31^+^ECs and was identical to the area of NG2^+^ cells (Figure 6a and b). On day 5 after injury, highly expressed Ninj1^+^ cells appeared in the mouse skin samples, some of which were around CD31^+^ ECs of the growing neovasculatures along the wound margins (Figure 5c). Similar observations were made for the immunostained human skin ulcer sections (Figure 5c). Moreover, costaining for NG2 and Ninj1 revealed that Ninj1 was coexpressed with NG2 along the wound margins of WT mice and humans (Figure 5d). Therefore, it is considered that Ninj1 expression is increased around blood vessels, especially in NG2^+^ cells during wound healing in mice and humans.

### Deletion of *Ninj1* in NG2^+^cells delays wound healing

**Figure 7:**
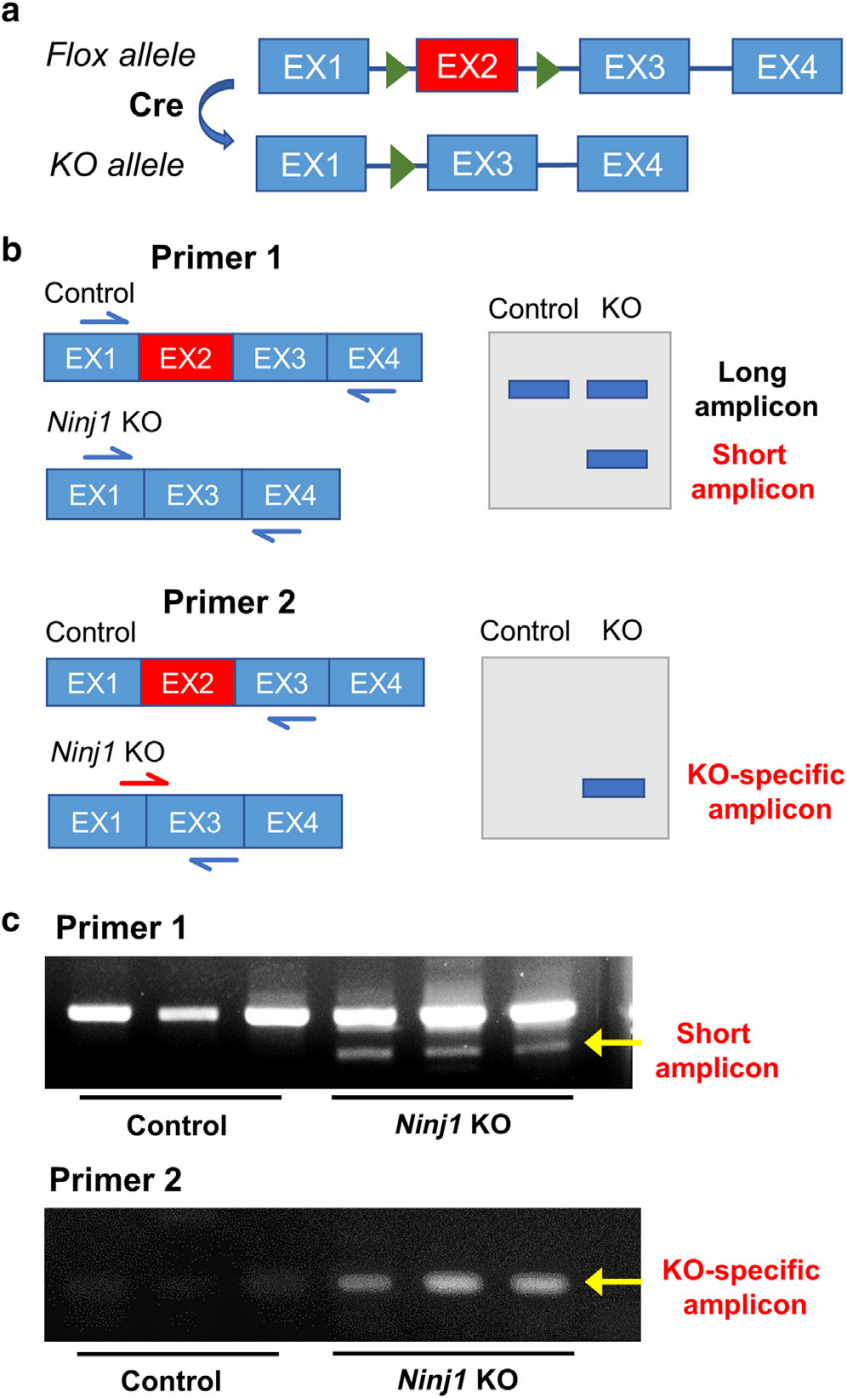
**PCR analysis for NG2-specific deletion of *Ninj1* gene.** (**a**) *Ninj1* consists of four exons. In *Ninj1*-KO mice, the second exon of *Ninj1* is subjected to NG2-specific deletion by the CreER-loxP system. (**b**) Two primer pairs were designed: primer1: forward/reverse primers for exon 1 and exon 4 of *Ninj1* and primer 2: forward primer at the exon1-exon3 junction. (**c**) Representative genomic PCR analysis. Using primer 1, a short amplicon was detected in *Ninj1*-KO mice, and using primer 2, KO-specific amplicons were detected only in *Ninj1*-KO mice. EX, exon; KO, knockout; Ninj1, Ninjurin-1.

**Figure 8:**
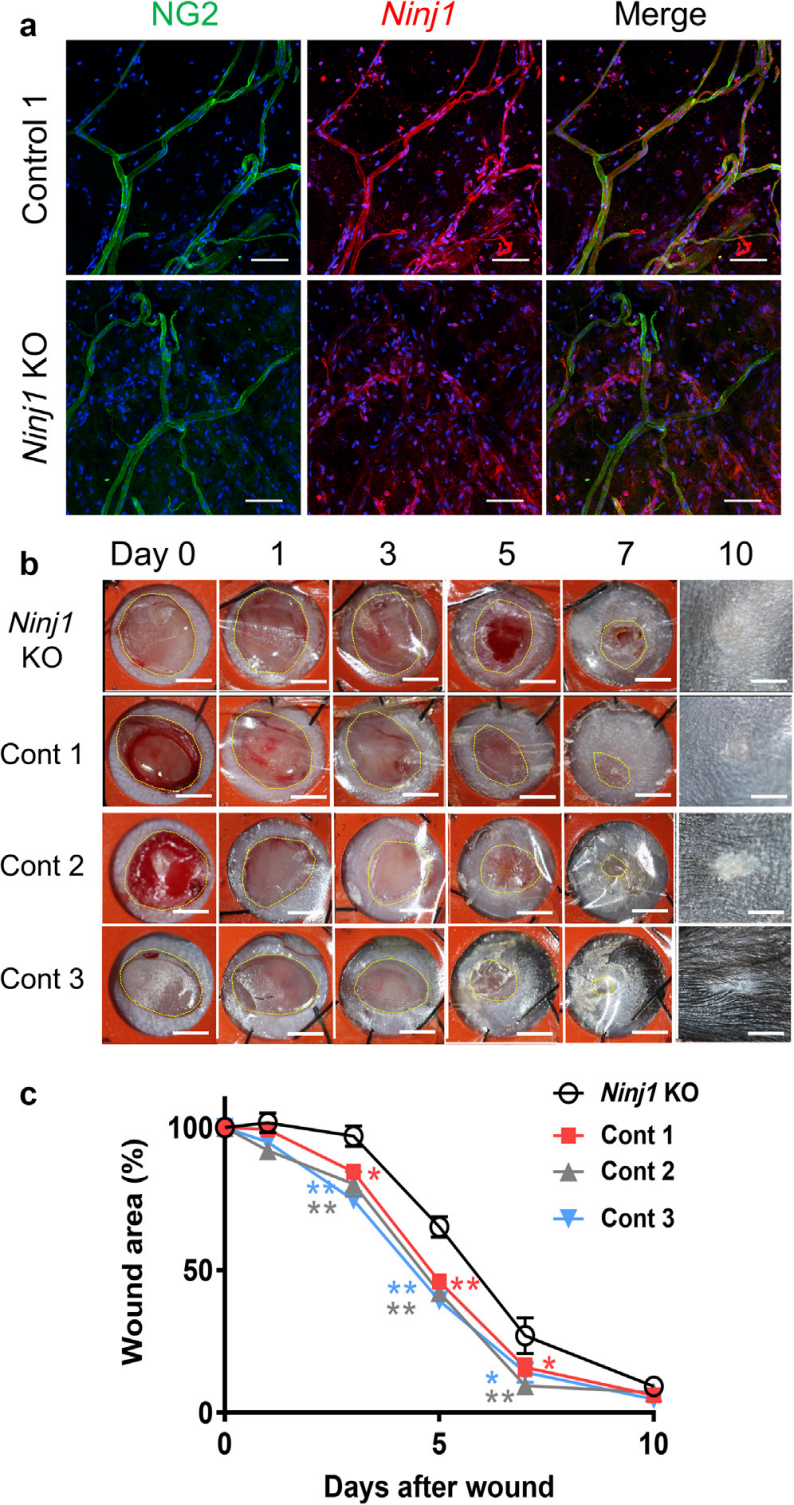
**Skin wound healing is delayed by NG2-specific deletion of *Ninj1* gene.***Ninj1* deletion was induced in pericytes by Tam treatment using NG2-CreER/Ninj1^loxP^ mice (*Ninj1* KO). Tam-treated Ninj1^loxp^, Tam-treated NG2-CreER, and Tam-untreated NG2-CreER/Ninj1^loxp^ mice designated as Conts 1, 2, and 3, respectively. (**a**) Whole-mount immunostaining for Ninj1 and NG2 of ear skin. (**b**) Representative photographs of wound skin in control and *Ninj1-*KO mice at indicated time points. (**c**) Percent ratio of wound area relative to the original wound area (wound area [%]) at each time point in control and *Ninj1*-KO mice. Data are expressed as mean ± SEM. ∗*P* < 0.05 and ∗∗*P* < 0.01 versus *Ninj1* KO; n = 5-8. Bars = 50 μm for **a** and 1 mm for **b**. Cont, control; KO, knockout; Ninj1, Ninjurin-1; Tam, tamoxifen.

To evaluate the impact of Ninj1 expression on the functions of PCs/VSMCs during wound healing, we induced *Ninj1* gene silencing in NG2^+^cells (*Ninj1* knockout [KO]) using tamoxifen (Tam) treatment before inflicting skin injury in the NG2CreER:Ninj1^loxP^ mice. We designated Tam-treated Ninj1^loxp^ and NG2-CreER mice as control groups 1 and 2, respectively, whereas Tam-untreated NG2-CreER: Ninj1^loxp^ mice formed the control group 3. Genetic recombination‒mediated *Ninj1* disruption was confirmed by PCR genomic analysis (Figure 7), and whole-mount‒immunostained ear skin samples showed Ninj1expression in NG2^+^ PCs/VSMCs of control mice but not in *Ninj1*-KO mice (Figure 8a).

Full-thickness skin wounds were created on the back of *Ninj1*-KO and control mice. The areas of the wounds were captured by digital imaging at various time points after injury (Figure 8b). On day 10 after injury, the wound areas appeared smaller, and the skin defect area had completely closed. Although the time course of wound healing was mostly identical among the three control groups, the wound areas were significantly larger in *Ninj1*-KO mice on days 3, 5, and 7 than in the three control groups (Figure 8c). These results indicate that skin wound healing is delayed when Ninj1 expression is suppressed in PCs/VSMCs during wound healing.

### Delayed re-epithelialization of wound skin by deletion of *Ninj1* in NG2^+^cells

**Figure 9:**
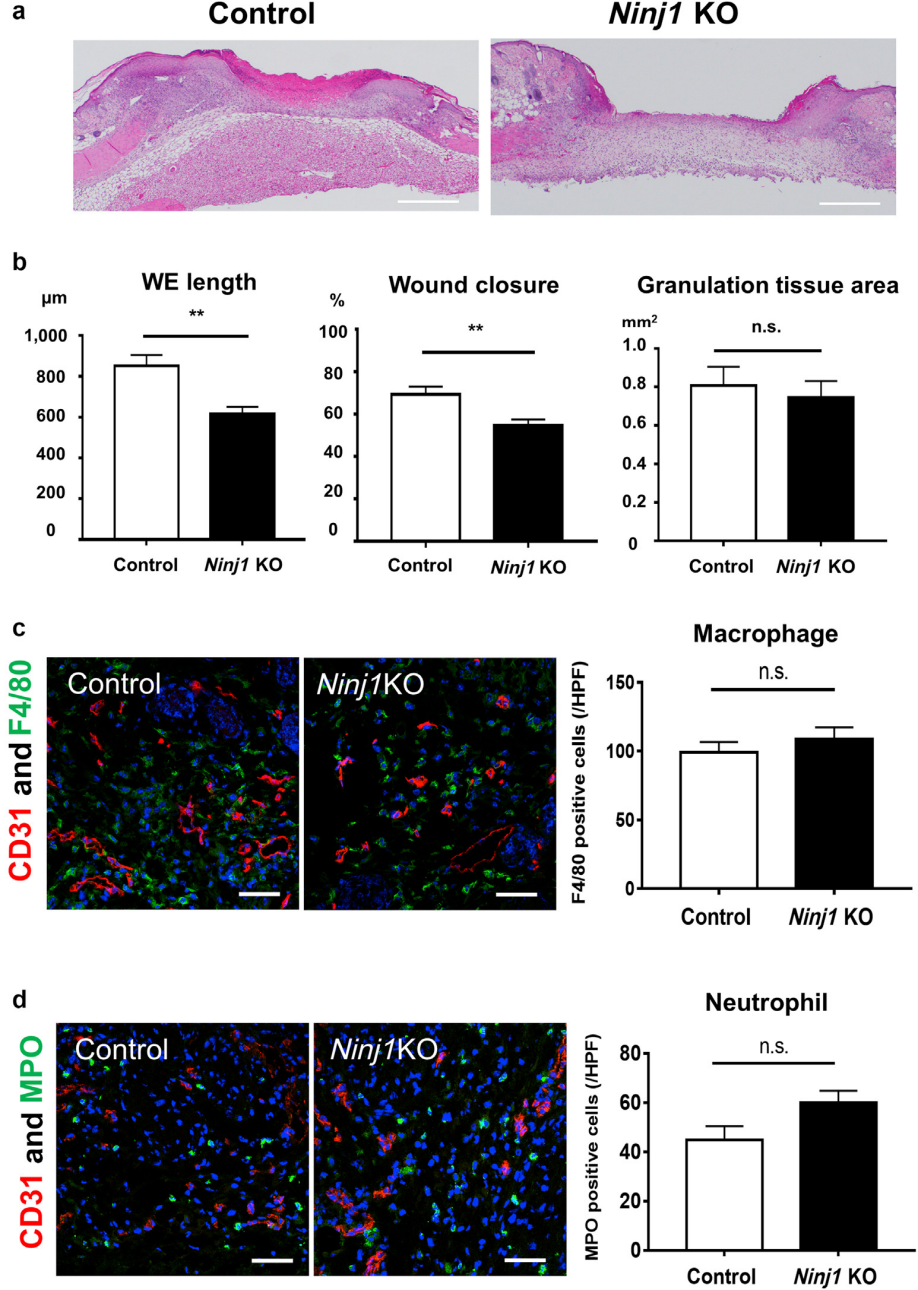
**NG2-specific *Ninj1* KO reduces re-epithelialization of wound skin during healing.** (**a**) Representative H&E images on day-5 skin wounds of control 1 and NG2-specific *Ninj1-*KO mice. Bars = 500 μm. (**b**) Wound length, wound closure, and granulation tissue area are evaluated in control and NG2-specific *Ninj1*-KO mice. (**c, d**) Representative images of inflammatory cell infiltration on day-5 wound skin sections of *Ninj1*-KO and control 1 mouse. (**c**) F4/80^+^ macrophages and (**d**) MPO^+^ neutrophils were costained with CD31 to evaluate inflammatory cell infiltration in the wound margin. Data are expressed as mean ± SEM. ∗∗*P* < 0.01; n = 9 (for **a** and **b**) and 4 (**c** and **d**). KO, knockout; MPO, myeloperoxidase; Ninj1, Ninjurin-1; n.s., not significant; WE, wound epithelium.

**Figure 10:**
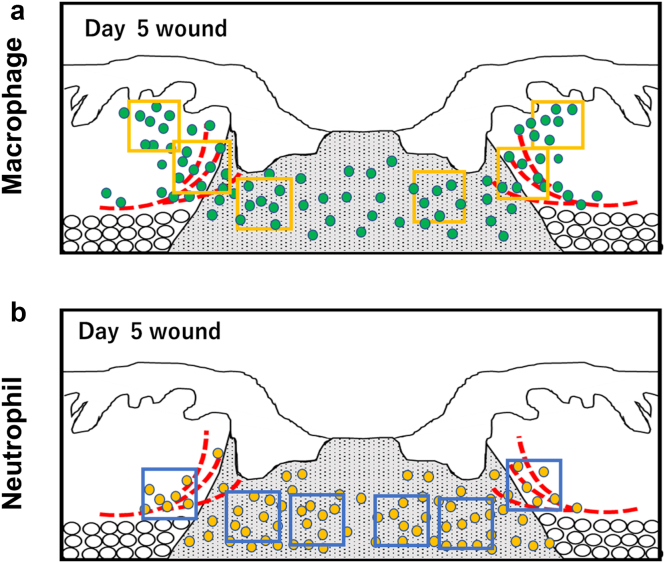
**Effects of NG2-specific *Ninj1* deletion on infiltrating inflammatory cells in wound skin.** Evaluation of inflammatory cells infiltrated in wound skin sections of NG2-specific *Ninj1*-KO and control mice. Immunostaining for CD31 and F4/80 or MPO was performed using day-5 wound sections. Schematic drawing showing the distribution of (**a**) F4/80^+^ macrophages and (**b**) MPO^+^ neutrophils infiltrated into the wound skin. The red dotted lines indicate new blood vessels at the wound margin. Six HPFs were selected from hotspots with inflammatory cells. Macrophages infiltrated into the wound margin and granulation tissue. Four HPFs were selected from the wound margin, and two HPFs were selected from the granulation tissue area. Neutrophils were mostly infiltrated into the granulation tissue; accordingly, two HPFs were selected from the wound margin, and four HPFs were selected from the granulation tissue area. HPF, high-power field; KO, knockout; MPO, myeloperoxidase; Ninj1, Ninjurin-1.

To examine whether NG2-specific *Ninj1* deletion affects the re-epithelialization of skin wounds, we further histologically evaluated the wound epidermis (Figure 3a and b). On day 5 after wound injury, *Ninj1-*KO mice had significantly lower wound epithelium length and wound closure than control mice (Figure 9a and b). However, the formation of granulation tissue was not altered (Figure 9a and b). To evaluate the infiltration of inflammatory cells into the injury, F4/80^+^ macrophages and myeloperoxidase-positive neutrophils were counted using skin sections of *Ninj1*-KO and control 1 mice. F4/80^+^ macrophages infiltrated into the wound margin and granulation tissue, whereas myeloperoxidase-positive neutrophils were distributed mainly in the granulation tissue area (Figure 10). There was no statistically significant difference in the number of infiltrated inflammatory cells, macrophages, and neutrophils between *Ninj1*-KO and control 1 mice (Figure 9c and d).

### Impairment of vessel formation in NG2^+^cell‒specific *Ninj1*-KO mice during skin wound healing

**Figure 11:**
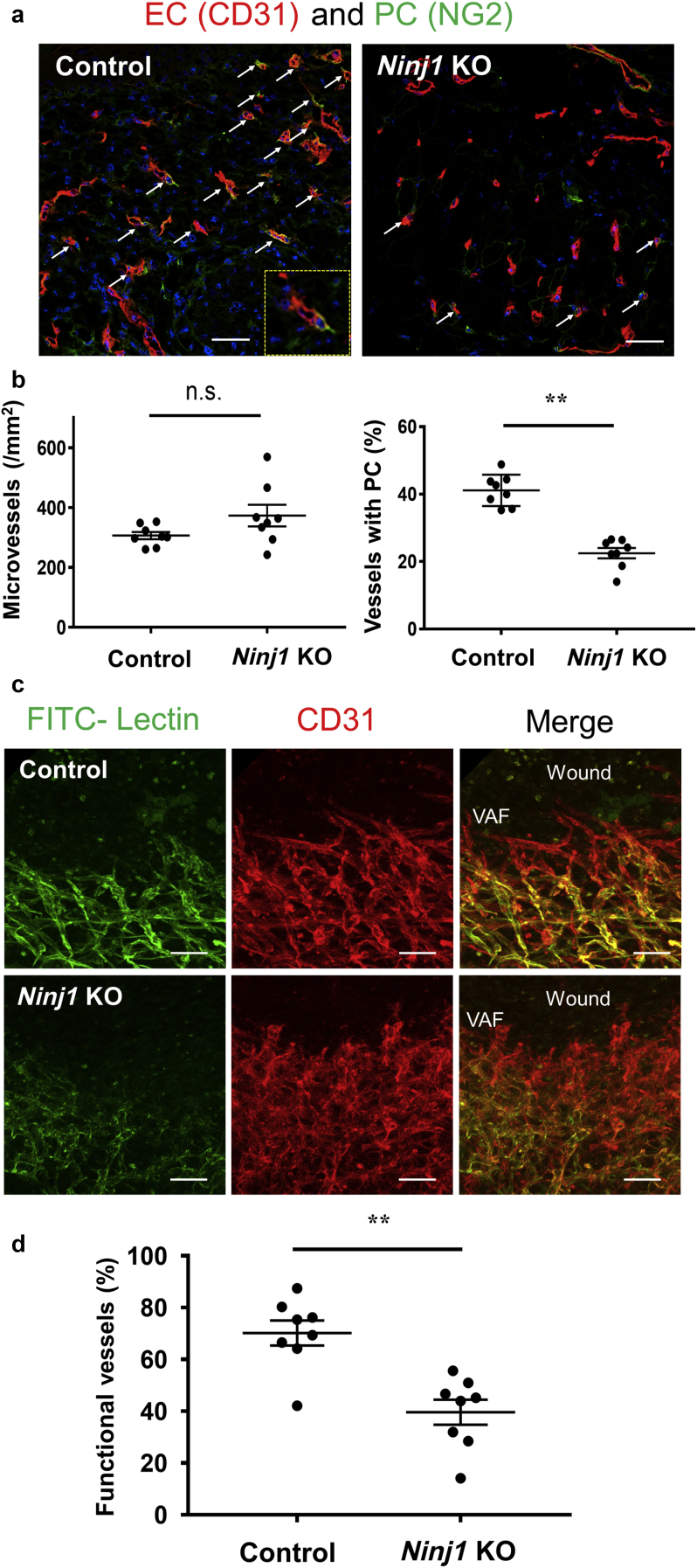
**NG2-specific deletion of gene encoding *Ninj1* impairs functional vessel formation during wound healing.** (**a**) Immunofluorescence staining for CD31 and NG2 in day-5 wound margins of *Ninj1-*KO and control 1 mice. Arrow indicates PC-associated vessels. (**b**) The estimated total number of CD31^+^ microvessels and ratio of PC-associated vessels to total microvessels. (**c**) On day 5 after wounding, functional vessels were stained with FITC-lectin, followed by whole-mount staining for CD31. Representative image of VAF of each mouse shown in three dimensions. (**d**) The ratio of functional vessels to the total number of microvessels compared between *Ninj1-*KO and control mice. Data are expressed as mean ± SEM. ∗∗*P* < 0.01; n = 4 (a, b), n = 8 (c, d). Bars = 50 μm. K knockout; Ninj1, Ninjurin-1; n.s., not significant; PC, pericyte; VAF, vascular advance front.

To explore the underlying mechanism of delayed wound healing in the case of *Ninj1* silencing, we observed the formation of new vessels within the wounded skin (Figure 3b and c). Microvessels were grown along the wound margins of both *Ninj1*-KO and control 1 mice (Figure 11a). The total number of microvessels (diameter < 10 μm) in this area did not differ significantly between *Ninj1*-KO and control mice (Figure 11b). However, double immunostaining of the skin sections for CD31 and NG2 on day 5 after wounding showed that *Ninj1*-KO mice had a significantly lower ratio of PCs-associated vessels to total vessels than that in the control group (Figure 11b). As shown in Figure 11c, functional, perfused vessels were stained with intravenous circulating FITC-lectin, and total microvessels were detected by EC-specific CD31 immunostaining. More than 70% of the microvessels were functional within the wound area of the control group, whereas in *Ninj1*-KO mice, the proportion of functional vessels was significantly low (Figure 11d).

### Lineage tracing of NG2^+^ cells during wound healing

Some PC populations may act as mesenchymal stem cells, thereby contributing to tissue remodeling and regeneration. Perivascular cells differentiate into myofibroblasts and contribute to the fibrosis of damaged organs (Kramann et al., 2015).

**Figure 12:**
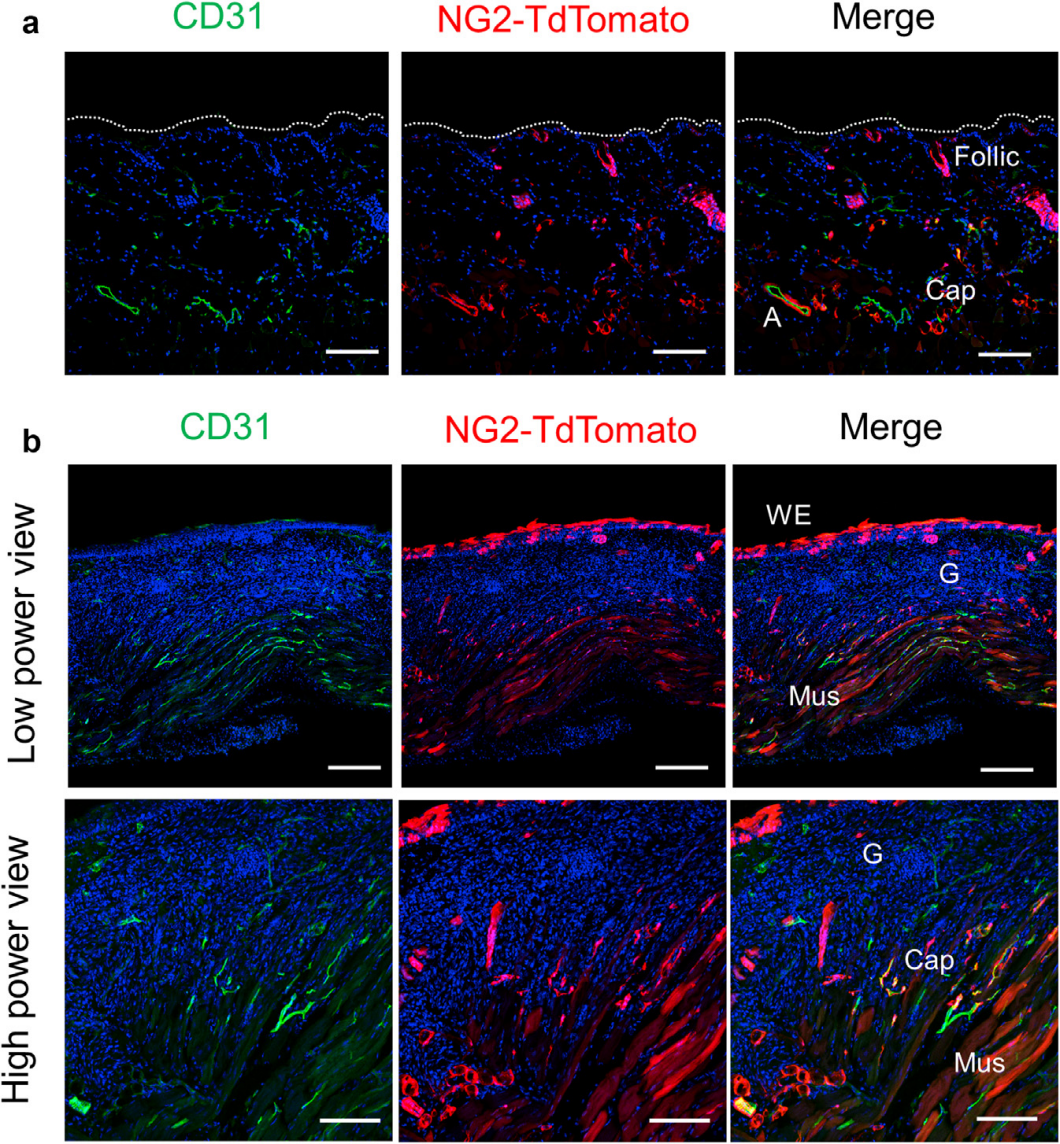
**Lineage tracing of NG2^**+**^ cells during wound healing.** (**a**) NG2^+^ cells were labeled by Tam treatment using NG2CreER:R26R-tdTomato mice before skin injury. Representative image represents immunostaining for CD31 in normal skin sections. TdTomato-expressing NG2^+^cells detected at Caps and Follic. (**b**) On day 5 after wounding, TdTomato-expressing cells were determined in Caps, WE, and Mus but not in G in the wound skin sections. Bars = 50 μm. Cap, capillary; Follic, follicle; G, granulation tissue; Mus, muscular fiber; Tam, tamoxifen; WE, wound epithelium.

To determine the contribution of NG2^+^ cells in wound healing, we used a genetic lineage tracing mouse model. For this, we labeled the NG2^+^ cells by Tam treatment using NG2CreERT:Rosa26tdTomato mice before skin injury. As shown in Figure 12a, red fluorescent protein (tdTomato)-expressing cells were observed at perivascular sites of the microvasculature in noninjured skin tissue in a pattern similar to that of NG2-DsRed mice (Figure 1). Moreover, on day 5 after injury, tdTomato-expressing cells were present not only at the PCs of growing vasculature but also at the regenerated KC layer, which covered the granulation tissue, and at the skeletal muscle fibers in the deeper dermis. However, there were no tdTomato signals within the granulation tissue (Figure 12b). Hence, NG2^+^cells act as KC and muscular fiber precursors during wound healing but do not contribute to the formation of granulation tissues.

## Discussion

Not only the early angiogenesis step, that is, EC sprouting, but also the later vascular maturation step is important for tissue regeneration followed by tissue damage. Previously, we had reported that Ninj1 in PCs plays an important role in vascular maturation to form functional microvessels under pathological conditions, such as ischemic hind limbs (Horiuchi et al., 2021; Minoshima et al., 2018). In this study, we investigated the role of Ninj1 in angiogenesis during wound healing. We showed that Ninj1 is expressed in perivascular cells of cutaneous blood vessels, and its expression increases temporarily in response to skin injury. When *Ninj1* expression is inhibited using NG2^+^cell‒specific Ninj1-deficient mice, microvasculatures growing in the wound margin area do not mature into functional vessels, and there is a lack of PC association with the vessels. Moreover, deletion of NG2^+^cell‒specific *Ninj1* significantly delays wound healing. Therefore, we revealed that Ninj1 is involved in prompt cutaneous wound healing by mediating appropriate angiogenesis to form matured microvessels.

Ninj1 was detected in the PCs and VSMCs of capillaries and arterioles, respectively, in the noninjured skin tissues (Figure 4). We successfully induced *Ninj1* gene silencing and inhibited Ninj1 protein expression in the PCs/VSMCs of skin tissue (Figure 8a). According to our previous studies, *Ninj1* deletion does not affect the vascular function and/or structure of pre-existing vessels, but it attenuates the maturation of neovasculature by inhibiting the interaction between PCs and sprouted EC tubes (Horiuchi et al., 2021; Minoshima et al., 2018). Ninj1 expression is induced by several pathophysiological conditions, including inflammation and ischemia (Matsuki et al., 2015). Similarly, we observed a temporary increase in *Ninj1* expression in injured skin tissues, particularly at the perivascular sites (Figure 5). In addition, NG2^+^ cell‒specific *Ninj1* deletion led to the formation of immature neovasculature in the regenerative wound margins. This effect is associated with a reduction in the proportion of PC-associated microvessels, whereas the total number of microvessels remains unchanged (Figure 11).

On the basis of markers and morphology, PCs have heterogeneous cell populations and can alter their phenotypes under pathogenic conditions (Vanlandewijck et al., 2018). Morikawa and Ezaki (2011) reported that PCs differ in their phenotype during wound healing in mouse skin, according to the stage of angiogenesis. At the leading edge of the growing microvessels in the wound margins (vascular-advancing front), PDGFRβ^+^NG2^‒^PCs promote angiogenesis during the early stages, whereas in the later stage, PDGFRβ^+^ NG2^+^PCs stabilize the neovasculature to form mature functional vessels. Consistent with this concept, lectin-negative nonfunctional vessels were observed at the vascular-advancing front, followed by lectin-positive functional vessels in the wound margin area. Therefore, on *Ninj1* deletion in NG2^+^ PCs, only the functional vessels were attenuated, whereas the total number of neovessels was not altered in wound skin (Figure 11). Microvessels were observed in the granulation area (Figure 9c and d), but no microvessels linked with NG2^+^ PCs were detected (Figure 12), suggesting that microvessels within the granulation tissues are immature PC free or NG2-negative PCs-associated microvessels. Thus, we were unable to evaluate the role of angiogenesis in the formation of granulation tissues using NG2^+^cell‒specific *Ninj1*-KO mouse model. The formation of granulation area was not altered (Figure 9). Impairment of vascular maturation induces blood circulatory disorder through nonfunctional microvessel formation (Horiuchi et al., 2021; Minoshima et al., 2018). Therefore, the consequent problems might disturb the regeneration of damaged skin tissues. *Ninj1* KO in NG2^+^ cells significantly delays wound healing, attenuation of wound epithelium growth, and wound closure.

Disorders of vascular maturation also induce persistent inflammation in regenerative tissues by exudation of blood cells, including inflammatory cells from vessels (Horiuchi et al., 2021), which contributes to delayed wound healing. Although we observed the infiltration of inflammatory cells at relatively acute phase, 5 days after, skin injury was not altered (Figure 9). The timing of infiltration might be altered by the kinds of inflammatory cells. Further study to estimate the infiltration of inflammatory cells at various times would be required to test the possibility of the contribution of inflammatory cells in delayed wound healing.

Fibroblasts, a major cellular component of connective tissue, are crucial for maintaining the extracellular matrix in normal and injured tissues. In the dermis, fibroblasts migrate into the granulation tissue, initiate collagen synthesis, and differentiate into myofibroblasts, which in turn generate force-inducing wound closure (Diaz-Flores et al., 2009). There is emerging evidence that PCs function as multipotent mesenchymal stromal cells that serve as a source of regenerating cells, including adipocytes and skeletal muscle cells, in response to tissue injury (Cathery et al., 2018; Kano et al., 2020). PCs can differentiate into myofibroblasts that support fibrosis in several organs, including kidneys and skin (Dulauroy et al., 2012; Humphreys et al., 2010). Moreover, dermal PCs are a potential source of myofibroblasts that mediate fibrosis during wound healing (Dulmovits and Herman, 2012; Greenhalgh et al., 2015). Alternatively, PCs deposit extracellular matrix, thereby promoting epidermal regeneration (Paquet-Fifield et al., 2009). Lineage-tracing experiments showed that NG2^+^ cells did not contribute to the formation of scar or granulation tissues but differentiated into KCs and deep dermis muscle fibers (Figure 12). In addition, granular formation was not affected by *Ninj1* KO (Figure 9). Therefore, *Ninj1* in NG2^+^ cells may not contribute to the supply of myofibroblasts and the formation of granular tissues during wound healing. We previously reported that some populations of NG2^+^ PCs have multipotency to differentiate into myocytes and contribute to the regeneration of myofibers (Kano et al., 2020). There is a possibility that deletion of *Ninj1* affects dermal muscular regeneration, which also involves wound contraction during the wound healing process. It is required to examine whether Ninj1 in NG2^+^cells regulate their myogenic potency.

**Figure 13:**
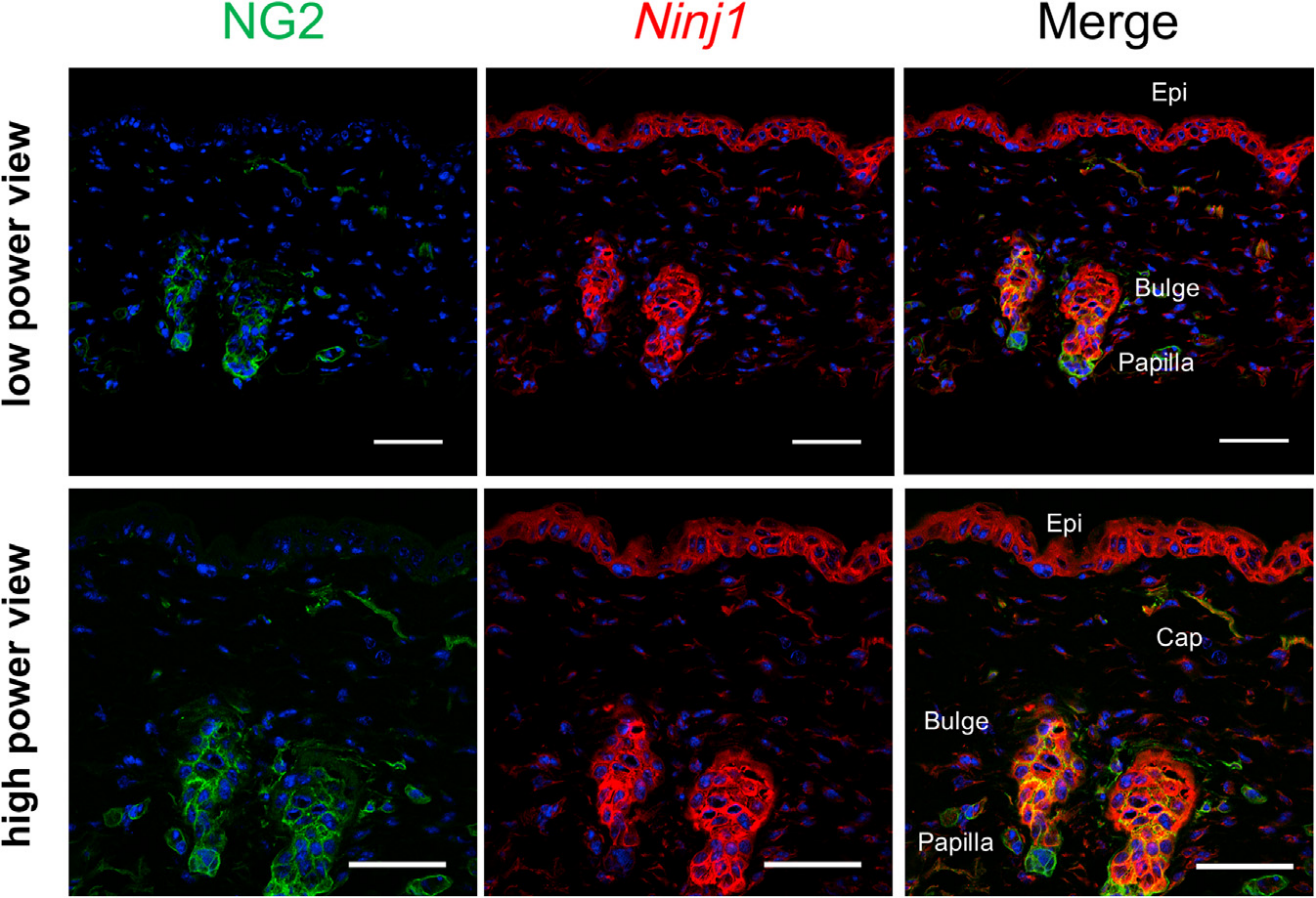
**The expression of Ninj1 in epidermis.** The expression of NG2 and Ninj1 in cutaneous hair follicles. Double immunostaining for NG2 and Ninj1 in normal skin sections of wild-type mice. NG2 and Ninj1 are coexpressed in Caps, and their expression is also partially consistent in the bulge region of the hair follicle. Bars = 50 μm. Bulge represents the bulge region. Papilla represents dermal papilla. Cap, capillary; Epi, epithelium; Ninj1, Ninjurin-1.

Although NG2 is utilized as a PC marker in most peripheral tissues, it is also expressed in stem cells in the cutaneous hair follicle bulge region, which contribute to hair as well as KC regeneration (Kadoya et al., 2008). Lineage-tracing studies showed that NG2^+^ cells contribute to the regeneration of the KC layer (Figure 12b). Moreover, Ninj1 is expressed in NG2^+^ cells, particularly at the bulge region of the hair follicle (Figure 13). Therefore, it is postulated that Ninj1 in NG2^+^ cells mediates wound healing through the function of follicle stem cells in addition to the effects on angiogenesis. Histological evaluation of wound epidermis showed that the formation of wound epidermis and subsequent closure by re-epithelialization were attenuated in *Ninj1* KO (Figure 9). In the future, it would be necessary to examine whether the delay in re-epithelialization is due to the direct effect of hair follicle cells on KC differentiation and re-epithelialization.

In conclusion, we show a previously unreported role of Ninj1 in prompt wound healing by mediating the maturation of neovessels in regenerative skin tissues. Notably, the increased incidence of diabetes mellitus is a global epidemic affecting national health in underdeveloped and developed countries. A severe complication of this disease is the occurrence of intractable skin wounds or ulcerations. Although the pathophysiology of diabetic wound healing is not completely understood, it is well-documented that microangiopathy is fundamental for diabetic disorders in the end organs, and one of the typical features of diabetic mellitus‒related microangiopathy is the abnormality in microvascular PCs (van Dijk et al., 2015). Therefore, this study provides valuable insight into microvascular maturation and its regulatory molecules, thereby suggesting therapeutic targets for such intractable wounds.

## Materials and Methods

### Animals

All experiments involving mice were performed according to the protocols approved by the Animal Care and Use Committee of Asahikawa Medical University (approval number R3-012). Animals, including WT (C57BL/6) and NG2-DsRed (female, aged 8‒12 weeks) mice, were maintained in a temperature- and light-controlled facility and were fed on normal chow. The NG2CreER:R26R-tdTomato and NG2CreER:Ninj1loxP mice (female, aged 10‒12 weeks) were used for genetic lineage tracing and development of KO mice, as described in previous studies (Minoshima et al., 2018; Tomita et al., 2019). Thereafter, Tam (Sigma-Aldrich, St. Louis, MO) was administered intraperitoneally to these mice at a daily dose of 100 μl (20 mg/ml in corn oil) for 5 consecutive days. After 1 week of Tam treatment, the mice were subjected to skin injury operation.

### Wound healing model

All surgical procedures were performed under 1.5–2.0% isoflurane inhalation anesthesia. The day before the wound creation, the mice’s back hair was plucked, and a depilatory cream was applied. Subsequently, two full-thickness skin wounds were created on the back of each mouse by disrupting the epidermis and deeper dermal layers using a 3-mm punch biopsy tool (Maruho, Osaka, Japan). To prevent wound contraction, a round silicone splint (inner diameter of 5 mm, outer diameter of 12 mm, Sigma-Aldrich) was placed over the ulcer and fixed using a cyanoacrylate adhesive (Konishi, Osaka, Japan) and interrupted 5‒0 nylon sutures (Kawano, Chiba, Japan). All wounds were covered with Tegaderm dressing (3M, Saint Paul, MN) to maintain a moist environment, and bandages (Careleaves, Nichiban, Tokyo) were wrapped around the abdomen to prevent the mice from scratching the wound (Galiano et al., 2004). Wound area size was digitally quantified on days 1, 3, 5, 7, and 10 after surgery using ImageJ software (National Institutes of Health, Bethesda, MD) and expressed as a percentage of the initial wound area (as calculated on day 0). Wound area reduction rate was described as the percentage from day 0 area of each lesion.

### Human subjects

Human subjects with normal skin (control) and wound skin at the Asahikawa Medical University Hospital participated in this study. For histological analysis, we collected skin samples from three patients with wound skin and three patients with benign skin tumors (lipoma), from which excess skin samples during the operation were used as healthy control. Human wound skin tissue samples were collected after receiving written informed consent from the subjects, according to protocols approved by the local ethical committee and the institutional review board of Asahikawa Medical University and in accordance with the 1964 Declaration of Helsinki. Histological analysis was performed for the samples procured from six subjects.

### Immunohistochemical and histological analyses

Histological assessment was performed on day 0 and days 1, 3, 5, and 10 after injury. Tissue samples were collected using a 5-mm punch biopsy tool (Maruho). One half of the collected specimen was embedded in an optimal cutting temperature compound (Sakura Finetek, Torrance, CA) for immunohistochemical analysis. The other half specimen was fixed with 4% paraformaldehyde for histological analysis. The tissue cross-sections (5-μm thickness) were fixed on glass plates with cold acetone. Thereafter, the target proteins were detected by immunohistochemical staining using the following primary antibodies (Table 1): anti-Ninj1 (ab213695, Abcam, Cambridge, United Kingdom), anti-CD31 (550274, BD Biosciences, Franklin Lakes, NJ), anti-NG2 (130-097-455, Miltenyi Biotec, Bergisch Gladbach, Germany), anti-PDGFRβ (AF1042, R&D systems, Minneapolis, MN), anti-F4/80 (123101, BioLegend, San Diego, CA), and antimyeloperoxidase (PA5-16672, Invitrogen, Carlsbad, CA), followed by Alexa 488‒, 568‒, and 594‒conjugated secondary antibodies (Invitrogen). Nuclei were counterstained with DAPI (D1306, Invitrogen). All immunofluorescent images shown in the figures are representative images (n = 3).

**Table 1:** Primary and Secondary Antibody List for Immunofluorescence and Western Blotting

Antibody	Product Description	Dilution	Source
Immunofluorescence			
Primary			
Ninjurin1 (ab213695 )	Rabbit, polyclonal	1:200	Abcam, Cambridge, UK
CD31 (550274)	Rat, monoclonal	1:200	BD Biosciences, Franklin Lakes, NJ
CD31 (AF3628)	Goat, polyclonal	1:400	R & D Systems, Minneapolis, MN
CD31 (M0823)	Mouse, monoclonal	1:200	Dako, Glostrup, Denmark
NG2 (130-097-455)	Rat, monoclonal	1:50	Miltery Biotec, Bergisch Gladbach, Germany
NG2 (14-6504-82)	Mouse, monoclonal	1:100	Invitrogen, Carlsbad, CA
PDGFR-beta (AF1042)	Goat, polyclonal	1:400	R & D Systems
F4/80 (123101)	Rat, monoclonal	1:200	BioLegend, San Diego, CA
MPO (PA5-16672)	Rabbit, polyclonal	1:100	Invitrogen, Carlsbad, CA
Rabbit IgG (ab171870)	Rabbit, polyclonal	1:200	Abcam
normal mouse IgG (sc-2025)	Mouse, polyclonal	1:200	Santa Cruz Biotechnology, Dallas, TX
Secondary			
Alexa Fluor 488 IgG	Goat, antimouse	1:1,000	Thermo Fisher Scientific, Waltham, MA
Alexa Fluor 488 IgG	Donkey, antirat	1:1,000	Thermo Fisher Scientific
Alexa Fluor 568 IgG	Goat, antirabbit	1:1,000	Thermo Fisher Scientific
Alexa Fluor 594 IgG	Donkey, antigoat	1:1,000	Thermo Fisher Scientific
Western blotting			
Primary			
Ninj1 (sc-136295)	Mouse, monoclonal	1:200	Santa Cruz, Dallas, TX
GAPDH (GTX28245)	Mouse, monoclonal	1:2,000	GeneTex, Los Angeles, CA
Secondary			
anti-mouse IgG, HRP-linked whole Ab	Sheep, antimouse	1:2,000	Cytiva, Marlborough, MA

Abbreviations: Ab, antibody; HRP, horseradish peroxidase; MPO, myeloperoxidase; UK, United Kingdom.

Fluorescence images were detected with a confocal fluorescence microscope (FV1000D, Olympus, Tokyo, Japan and BZ-X700, Keyence, Osaka, Japan). To evaluate the number of microvessels covered with PCs, we detected the CD31^+^ ECs and the ECs colocalized with NG2^+^ PCs in the skin tissue sections. In each mouse, we analyzed four selected fields (approximately 0.35 mm^2^ area, ×200 magnification) of wound margin area. The total number of microvessels was measured per mm^2^ of the observed field, and the number of PC-associated microvessels was expressed as a percentage (%) of the total microvessels per field.

For histological analysis, sections were deparaffinized and rehydrated for histological and immunohistochemical staining. Sections were stained with H&E for evaluation of morphology. Wound length and wound epithelium length are depicted in Figure 3 (Dahlhoff et al., 2017). Wound length was determined by the measurement of the length between the wound edges. The wound closure is calculated as a percentage of wound epithelium length to the wound length. The area of granulation tissue was evaluated by α-smooth muscle actin staining (M0851, Dako, Glostrup, Denmark). All measurements for histopathological analysis were performed using ImageJ software. Histopathological analyses were performed using the indicated number of mice.

### Whole-mount vascular imaging

To visualize the blood-circulating vessels, 350 μl of FITC-labeled Griffonia simplicifolia lectin (FL-1101, Vector Laboratories, Burlingame, CA) (100 μg/ml PBS) was administered into the tail veins of the mice under anesthesia. Subsequently, the mice were fixed by perfusion through the left ventricle with 4% paraformaldehyde in PBS (pH: 7.0) 15 minutes after the injection, thereby allowing the lectin to circulate into the entire vasculature. We collected ear skin tissues according to previous methods (Yamazaki et al., 2018). To collect the ear skin tissue, depilatory cream was applied externally to the pinna of the mouse for 1 minute, after which the dorsal side of the skin was peeled off and fixed with 4% paraformaldehyde or cold acetone. The wound skin was also collected using a 5-mm punch biopsy tool, fixed by immersion in 4% paraformaldehyde for 1 hour at 37 °C, and incubated with blocking buffer (3% BSA and 1% Triton-X 100 in PBS) for 1 day. Skin tissues were incubated with the earlier-mentioned primary antibodies in dilution buffer (1% BSA and 0.2% Triton-X 100 in PBS) at 4 °C for 48 hours. Thereafter, the samples were washed three times with washing buffer (3% sodium chloride and 0.2% Triton-X in PBS) and immersed in 400 μl of specific secondary antibodies at 4 °C for 24 hours.

To observe a 3D image of the skin tissue, the samples were clarified by treatment with RapiClear reagent (RC147001, SunJin Lab, Hsinchu City, Taiwan) for 24 hours at 37 °C and imaged using a confocal fluorescence microscope (FV1000D Olympus and BZ-X700 Keyence). To analyze the formation of functional blood vessels during wound healing, FITC-lectin‒stained vessels and CD31-immunostained vessels were observed in 3D, as described previously (Minoshima et al., 2018). In each skin sample, five high-power field (×400 magnification) areas were randomly selected along the wound margin and observed using Z-stack images (15 serial slides in 10 μm steps). Finally, we calculated the ratio of the area of blood-circulating, functional vessels to the area of the total vessels in each of our observed fields.

### RT-qPCR analysis

Total RNA was isolated from the mouse skin samples using the RNeasy Mini Kit (Qiagen, Hilden, Germany), and cDNA was synthesized using iScript cDNA Synthesis Kit (Bio-Rad Laboratory, Hercules, CA). Quantitative real-time RT-PCR was performed in triplicate using TaqMan Universal PCR Master Mix (Thermo Fisher Scientific, Waltham, MA) on a LightCycler 480 System (Roche Diagnostics, Basel, Switzerland). Fluorogenic probes and primers were used to detect Ninj1 (Mm00479014_m1) in mouse samples. Absolute threshold values (Ct values) were determined using SDS software 2.1 (Applied Biosystems, Waltham, MA). Fold changes in target gene expression were calculated using the formula ΔCt = Ct target – CT control and were normalized to that of *Gapdh* expression using the published comparative 2-ΔΔCt.

### Western blot analysis

The frozen skin tissue samples were dissolved in RIPA buffer (Wako, Osaka, Japan) containing a protease inhibitor cocktail (Sigma-Aldrich, Darmstadt, Germany), homogenized using Bead Mill 24 (Thermo Fisher Scientific), and sonicated. The solubilized sample was collected by centrifugation at 16,900*g* for 10 minutes. After adjusting the protein concentration (1 ng/ml) using a bicinchoninic acid protein assay kit (Pierce, Rockford, IL), the samples (20 μg/lane) were fractionated by SDS-PAGE and transferred to Hybond-PVDF membranes (Amersham Bioscience, Piscataway, NJ). The blotted membranes were blocked with Tris-buffered saline containing 0.1% Tween-20 and 10% skimmed milk and subsequently incubated overnight at 4 °C with anti-Ninj1 or anti-GAPDH antibodies (Table 1). The target protein bands were visualized using horseradish peroxidase‒conjugated secondary antibodies and quantified using ImageQuant LAS500 (GE Healthcare Life Science, Chicago, IL). ImageJ program was used for densitometry analysis.

### Statistical analysis

Experimental data are presented as mean ± SEM unless otherwise noted. Student *t*-test was applied to analyze the differences between two groups. One-way ANOVA with Dunnett’s multicomparison test was applied to analyze differences among more than two groups (GraphPad Prism 7.00, GraphPad Software, San Diego, CA). Statistical significance was set at *P* < 0.05.

### Data availability statement

Datasets related to this article can be found at https://data.mendeley.com/datasets/6vx4jy4b88/2, hosted at Mendeley.

## ORCIDs

Risa Matsuo: http://orcid.org/0000-0002-1486-5369

Mari Kishibe: http://orcid.org/0000-0002-2966-1756

Kiwamu Horiuchi: http://orcid.org/0000-0002-0126-8813

Kohei Kano: http://orcid.org/0000-0002-0223-5781

Takamitsu Tatsukawa: http://orcid.org/0000-0001-5791-6579

Taiki Hayasaka: http://orcid.org/0000-0002-2206-8562

Maki Kabara: http://orcid.org/0000-0002-7841-8824

Shin Iinuma: http://orcid.org/0000-0002-3673-0932

Ryoji Eguchi: http://orcid.org/0000-0002-2417-7578

Satomi Igawa: http://orcid.org/0000-0001-9483-2526

Naoyuki Hasebe: http://orcid.org/0000-0003-1395-4412

Akemi Ishida-Yamamoto: http://orcid.org/0000-0002-3104-102X

Jun-ichi Kawabe: http://orcid.org/0000-0002-4837-6598

## Author Contributions

Conceptualization: MKi, NH, AIY, JK; Formal Analysis: RM, MKi, SIi, SIg, AIY, JK; Funding Acquisition: NH, AIY, JK; Investigation: RM, MKi, KH, KK, TT, TH, SIi; Methodology: RM, MKi, KH, KK, TT, TH, MKa, SIi, RE, SIg, JK; Project Administration: RM, AIY, JK; Resources: RM, MKi, KH, KK, TT, TH, MKa, SIi, RE, SIg, AIY, JK; Supervision: MKi, HN, AIY, JK; Validation: RM, MKi, KH, KK, TT, SIi, SIg, AIY, JK; Writing - Original Draft Preparation: RM, JK; Writing - Review and Editing: MKi, SIi, SIg, HN, AIY, JK

## Conflict of Interest

Satomi Igawa reports a conflict of interest with Maruho.
